# The tumour metabolism inhibitors GSAO and PENAO react with cysteines 57 and 257 of mitochondrial adenine nucleotide translocase

**DOI:** 10.1186/1475-2867-12-11

**Published:** 2012-03-26

**Authors:** Danielle Park, Joyce Chiu, Gabriel G Perrone, Pierre J Dilda, Philip J Hogg

**Affiliations:** 1 Lowy Cancer Research Centre & Prince of Wales Clinical School, University of New South Wales, Sydney 2052, Australia; 2 Ramaciotti Centre for Gene Function Analysis, School of Biotechnology and Biomolecular Sciences, University of New South Wales, Sydney 2052, Australia

**Keywords:** Tumour metabolism, Mitochondria, Adenine nucleotide translocase, GSAO, PENAO

## Abstract

**Background:**

GSAO (4-(N-(S-glutathionylacetyl)amino) phenylarsonous acid) and PENAO (4-(N-(S-penicillaminylacetyl)amino) phenylarsonous acid) are tumour metabolism inhibitors that target adenine nucleotide translocase (ANT) of the inner-mitochondrial membrane. Both compounds are currently being trialled in patients with solid tumours. The trivalent arsenical moiety of GSAO and PENAO reacts with two matrix facing cysteine residues of ANT, inactivating the transporter. This leads to proliferation arrest and death of tumour and tumour-supporting cells.

**Results:**

The two reactive ANT cysteine residues have been identified in this study by expressing cysteine mutants of human ANT1 in *Saccharomyces cerevisiae *and measuring interaction with the arsenical moiety of GSAO and PENAO. The arsenic atom of both compounds cross-links cysteine residues 57 and 257 of human ANT1.

**Conclusions:**

The sulphur atoms of these two cysteines are 20 Å apart in the crystal structures of ANT and the optimal spacing of cysteine thiolates for reaction with As (III) is 3-4 Å. This implies that a significant conformational change in ANT is required for the organoarsenicals to react with cysteines 57 and 257. This conformational change may relate to the selectivity of the compounds for proliferating cells.

## Background

Healthy cells mainly rely on oxidative phosphorylation to catabolise glucose, while cancer cells employ aerobic glycolysis to catabolise both glucose and glutamine [1]. Mitochondria coordinate the catabolism of glucose and glutamine in cancer cells so targeting this organelle has potential for the treatment of this disease. A promising molecular target is the hexokinase II-voltage dependent anion channel-adenine nucleotide translocase complex that spans the outer- and inner-mitochondrial membranes. This complex links glycolysis, oxidative phosphorylation and mitochondrial-mediated apoptosis in cancer cells.

The first step in glycolysis, conversion of glucose and ATP to glucose-6-phosphate (G-6-P) and ADP, is catalyzed by hexokinase and cancer cells mostly employ an isoform (HKII) that is bound to mitochondria via interaction with the outer-membrane voltage dependent anion channel (VDAC) [2-5]. VDAC is associated with inner-membrane adenine nucleotide translocase (ANT), which exchanges matrix ATP for cytosolic ADP across the inner-membrane [6]. ANT is thought to have two functions in cancer cells: it provides ATP to hexokinase II, to phosphorylate and trap glucose in the cell [1], and is a component of the mitochondrial permeability transition pore [6], which is involved in the permeability of the inner-mitochondrial membrane. Opening of this pore by inactivating ANT allows the equilibration of solutes <1500 Da in size across the inner-membrane. This leads to uncoupling of oxidative phosphorylation and increase in superoxide levels, loss of trans-membrane potential and decrease in oxygen consumption. These effects of ANT blockade result in proliferation arrest and mitochondrial-mediated apoptotic cell death [7].

**Figure 1:**
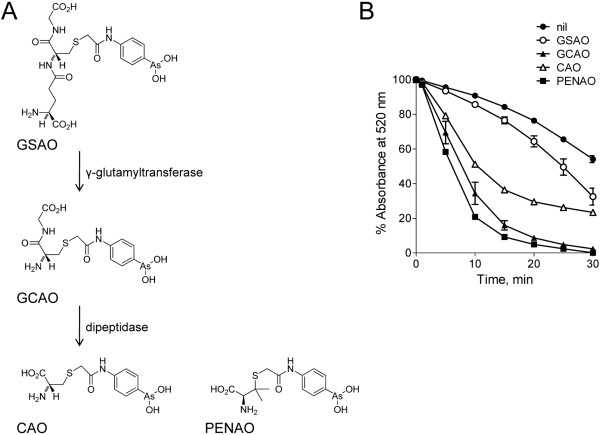
**GSAO, its metabolites GCAO and CAO, and PENAO trigger opening of the mitochondrial permeability transition pore**. **A**. Structures of GSAO, its metabolites, and PENAO. L-Glutamyltransferase catalyses the hydrolysis of the peptide bond between GSAO's L-glutamic and cysteine residues, while a dipeptidase hydrolyses the peptide bond between GCAO's glycine and cysteine residues. PENAO is a cysteine mimetic of CAO. **B**. Mitochondrial transition pore opening triggered by GSAO, GCAO, CAO and PENAO. Rat liver mitochondria were incubated with 100 μM of the different compounds and swelling measured by decrease in light scattering at 520 nm over 30 min. The data points and error bars are the mean ± SD from 4 experiments (performed in duplicate with two different mitochondrial preparations).

GSAO (4-(N-(S-glutathionylacetyl)amino) phenylarsonous acid) is an ANT inhibitor that is currently being trialled in a Phase I/IIa dose escalation study in patients with solid tumours refractory to standard therapy. The trivalent arsenical of GSAO reacts with ANT in angiogenic endothelial cells and inhibits tumour angiogenesis and tumour growth in mice [7]. Metabolism of GSAO at the cell surface is required for it to exert its anti-mitochondrial effect. GSAO is first cleaved by γ-glutamyltranspeptidase at the cell surface to produce GCAO (4-(N-(S-cysteinylglycylacetyl)amino) phenylarsonous acid) (Figure 1A). GCAO then enters the cell via an organic ion transporter and is further processed by dipeptidases to CAO (4-(N-(S-cysteinylacetyl)amino) phenylarsonous acid) in the cytosol [8]. CAO enters the mitochondrial matrix and reacts with ANT. A second generation ANT inhibitor, PENAO (4-(N-(S-penicillaminylacetyl)amino)phenylarsonous acid), has been designed to bypass the pro-drug processing and metabolism of GSAO [9]. PENAO is a cysteine mimetic of CAO (Figure 1A). PENAO accumulates in cells 85-fold faster than GSAO, which results in a 44-fold increased anti-proliferative activity and a ~20-fold increased anti-tumour efficacy in mice. In contrast to GSAO, PENAO targets both proliferating endothelial and tumour cells. A Phase I/IIa dose escalation study of PENAO in patients with solid tumours refractory to standard therapy is currently recruiting. The molecular mechanism of action of GSAO and PENAO was explored in this study by identifying the ANT residues that react with the compounds.

## Results and discussion

GSAO, its metabolites GCAO and CAO, and PENAO triggered permeability transition pore formation in rat liver mitochondria (Figure 1B). GCAO, CAO and PENAO were more efficacious than GSAO, possibly because of their faster entry into mitochondria due to their smaller size (see Figure 1A). This result indicates that the arsenic atom of all four compounds reacts with ANT. Trivalent arsenicals (As(III)) react with closely spaced protein thiols, forming stable, cyclic dithioarsinite complexes in which both sulphur atoms are bound to arsenic [10]. We next determined which two ANT cysteines are cross-linked by the compounds.

Modulating the expression of or mutagenesis of ANT in mammalian cells is not practical because any disruption of endogenous ANT leads to perturbation of mitochondrial function and apoptosis of the cell [11]. However, mammalian ANT can be expressed and function in yeast. *S. cerevisiae *possesses three homologues of mammalian ANT (Aac1p, Aac2p and Aac3p) and disruption of one or more of the genes encoding these proteins generates cells that are unable to grow using non-fermentable sources of carbon, such as glycerol and ethanol [12]. Mammalian ANT rescues this growth defect in yeast [13]. To probe the ANT cysteine residues that GSAO cross-links, human wild-type and cysteine mutant ANT1 was expressed in *S. cerevisiae *and binding of biotin-tagged GSAO and PENAO was measured.

**Figure 2:**
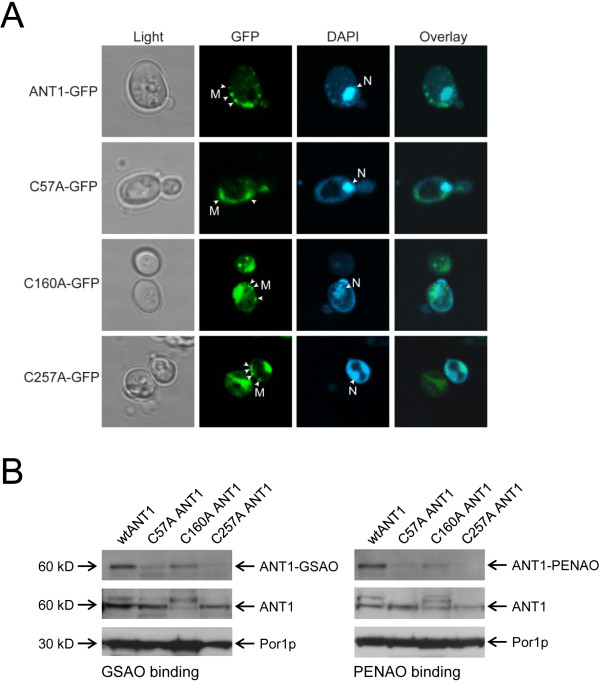
**The trivalent arsenical of GSAO and PENAO reacts with cysteines 57 and 257 of human ANT1**. **A**. Localisation of human ANT1-GFP in *S. cerevisiae*. Yeast cells expressing ANT1-GFP and the cysteine mutants were analysed by fluorescence microscopy to determine cellular localisation. Cells were also stained with DAPI to identify mitochondrial and nuclear DNA. Wild-type and ANT1 cysteine mutants co-localized with mitochondrial DNA. The C160A and C257A ANT1 mutants were also found in the cytoplasm, which likely represents incomplete or inefficient incorporation into mitochondria. White arrows indicate the mitochondria (M) or the nucleus (N). **B**. Mitochondria from *S. cerevisiae *expressing ANT1-GFP and the cysteine mutants were isolated and incubated with biotin-tagged GSAO or PENAO. The labelled proteins were collected on avidin-coated beads and bound ANT1 was detected by Western blot. GSAO- and PENAO-biotin bound to wild-type and C160A mutant ANT1 but not to C57A and C257A mutants. The human ANT1 and yeast Por1p in the mitochondrial preparations was blotted to show the input protein in the binding assay. The sizes the proteins in kDa is indicated at left. The results from two separate experiments are shown.

Human ANT1 and the cysteine mutants co-localized with mitochondrial DNA in the yeast cells, indicating that the proteins were processed correctly for mitochondrial insertion (Figure 2A). The C160A and C257A ANT1 mutants were also found in the cytoplasm, which likely represents incomplete or inefficient incorporation of these mutants into mitochondria. This observation was confirmed when the mitochondrial fractions from the yeast cells were blotted for ANT1. There was less of these mutant proteins in the mitochondria than for the wild-type and C57A mutant (Figure 2B). Biotin-tagged GSAO and PENAO clearly bound to wild-type ANT1 and the C160A mutant protein, despite lower levels of the mutant, but did not appreciably interact with the C57A or C257A ANT1 mutants (Figure 2B). These results imply that the arsenic atom of the peptide arsenicals cross-links cysteine residues 57 and 257 of ANT. All four isoforms of human ANT (1-4) contain the same three cysteine residues (residues 57, 160 and 257), so it is likely that the organoarsenicals will react with all of the human ANTs.

## Conclusions

**Figure 3:**
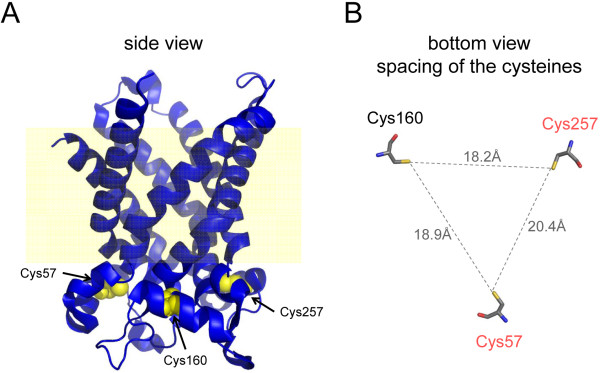
**Crystal structure of bovine ANT and spacing of the matrix facing cysteine thiols**. A side view of the structure [15] is shown in part A. The matrix facing cysteine residues are in yellow space-filling representation. The cysteine numbering is for the human protein. The corresponding bovine numbering is Cys56, Cys159 and Cys256. The yellow background represents the approximate position of the inner-mitochondrial membrane. The spacing of the three unpaired cysteine residues is shown in part B. The structures were generated from PDB 1OKC using PyMol [31].

The finding that GSAO and PENAO cross-links Cys57 and Cys257 of ANT is seemingly at odds with the observation that GSAO binding competes for alkylation of rat liver ANT by eosine-maleimide [7]. Eosine-maleimide alkylates Cys160 in ANT and blocks binding of the small arsenical, phenylarsine oxide [14]. It is clear that GSAO- and PENAO-biotin interacts with the C160A ANT1 mutant, though (Figure 2B). Cross-linking of Cys57 and Cys257 may sterically block alkylation of Cys160 by eosine-maleimide, which is a bulky compound with a molecular mass of 743 Da. Conversely, eosine-maleimide alkylation of Cys160 may block reaction of GSAO or phenylarsine oxide with Cys57 and Cys257. These scenarios are reasonable considering the close proximity of the cysteine thiols in the tertiary structure. The three matrix facing thiols are 18-20 Å apart in the crystal structures of bovine ANT [15,16] (Figure 3).

The optimal spacing of cysteine thiolates for reaction with As(III) is 3-4 Å [17,18]. This implies that a significant conformational change in ANT (~15 Å of movement) is required for GSAO and PENAO to react with cysteines 57 and 257. It is possible that the flexibility of the matrix facing loops relates to the selectivity of GSAO and PENAO for proliferating cells. The calcium within the mitochondrial matrix increases several-fold in proliferating cells [19]. GSAO and PENAO have little effect on growth quiescent cells [7,9] and the bovine ANT structure represents the calcium-free form of the transporter [15,16]. Calcium binding to ANT may influence its structure and interaction with As(III).

## Methods

GSAO [7], GCAO [8], CAO [8], PENAO [9] and biotin-tagged GSAO and PENAO [7] were produced and titrated as described.

### Expression of human ANT1 in *Saccharomyces cerevisiae*

For expression of human ANT1 in *S. cerevisiae*, the coding sequence of the first 26 amino acid residues from yeast Aac2p followed by the ANT1 coding sequence was PCR amplified from y2NHANT1/pRS314 [20] and gateway cloned into the yeast expression vector pAG416GAL-ccdB-EGFP [21]. In this system, ANT1 is expressed as a fusion protein with EGFP at the C-terminus under regulation of the *GAL1 *promoter, which is repressed by glucose [22]. The ANT1-EGFP construct has a calculated molecular weight of 62.5 kDa. The C57A, C160A and C257A mutants of ANT1 were made using the QuikChange XL Site-Directed Mutagenesis kit (Integrated Sciences) and their sequence was confirmed. The wild type ANT1 and mutant constructs were transformed into yeast strain BY4741 (*MAT*a, *his3Δ, leu2Δ, ura3Δ, met15Δ*) as described [23]. Yeast transformants were selected in synthetic complete media lacking uracil (SC-ura: 20 g/L glucose, 5 g/L ammonium sulphate, 1.7 g/L yeast nitrogen base, 10 mg/L adenine, 50 mg/L arginine, 80 mg/L aspartate, 20 mg/L histidine, 50 mg/L isoleucine, 100 mg/L leucine, 50 mg/L lysine, 20 mg/L methionine, 50 mg/L phenylalanine, 100 mg/L threonine, 100 mg/L tryptophan, 50 mg/L tyrosine and 140 mg/L valine). ANT1-GFP protein was expressed by culturing yeast cells to an optical density of 0.8 at 600 nm in SC-ura media containing raffinose (20 g/L) instead of glucose [24]. Cells were cultured for another 6 h with 0.2% galactose before imaging or harvesting the mitochondria.

### Localization of ANT1 protein in yeast cells

Cells expressing ANT1-GFP and its mutant forms were stained with the nucleic acid dye DAPI (5 μg/mL) to visualize both nuclear and mitochondrial DNA. DAPI has been mostly used to visualise mitochondria in yeast cells when the ANT1 homologues (AAC1, AAC2, AAC3) are over-expressed [25,26]. Over-expression of ANT1 leads to reduction of mitochondrial membrane potential, which compromises the effectiveness of membrane potential-sensing dyes such as Mitotracker or rhodamine 123 [27,28]. Live cells were examined using a Leica TCS SP5 inverted confocal laser scanning microscope. GFP was excited at 488 nm with fluorescence signals collected between 520-650 nm. DAPI was excited by multiphoton at 800 nm with fluorescence signals collected between 405-470 nm. Bright-field and fluorescent images were collected and processed using ImageJ software [29].

### Mitochondrial assays

Mitochondria were isolated from the livers of male Wistar rats [7] or BY4741 *S. cerevisiae *expressing ANT1 [30]. Swelling assays using rat liver mitochondria were performed as described [7]. *S. cerevisiae *mitochondria (1 mg/mL in 3 mM Hepes-KOH, pH 7 buffer containing 213 mM mannitol, 71 mM sucrose and 10 mM sodium succinate) were incubated with 10 μM biotin-tagged GSAO or PENAO for 1 h at room temperature on a rotating wheel. The mitochondria were washed 3 times with the Hepes buffer to remove the unreacted compounds, resuspended in 0.3 mL ice-cold 25 mM Tris, pH 7.4 buffer containing 140 mM NaCl, 2.7 mM KCl, 0.5% Triton X-100, 0.05% Tween 20, 3 M urea, 5 mM EDTA and protease inhibitor cocktail (Roche), and incubated with 30 μL streptavidin-dynabeads (Invitrogen) for 2 h at 4°C on a rotating wheel. The beads were washed 5 times with 25 mM Tris, pH 7.4 buffer containing 140 mM NaCl, 0.5% Triton X-100 and 3 M urea and then resuspended in SDS-PAGE loading buffer containing 20 mM dithiothreitol. Samples were heated at 90°C for 5 min and the eluates resolved on NuPAGE Novex 4-12% Bis-Tris Gel (Invitrogen) with MOPS running buffer and transferred to polyvinylidene difluoride membrane. ANT was detected by Western blot using 1:100 dilution of anti-human ANT polyclonal antibody Q-18 (Santa Cruz) and 1:1000 dilution of rabbit anti-goat peroxidase-conjugated antibodies (Dako). The ANT1 and yeast porin (Por1p) in the mitochondrial preparations was blotted to show the input protein in the binding assay. Por1p was detected using 1:5000 dilution of anti- Por1p monoclonal antibody (Invitrogen) and rabbit anti-mouse peroxidase-conjugated antibodies (Dako). Chemiluminescence films were analyzed using a GS-700 Imaging Densitometer and Multi-Analyst software (Bio-Rad, Hercules, California).

## Abbreviations

ANT: Adenine nucleotide translocase; CAO: 4-(N-(S-cysteinylacetyl)amino) phenylarsonous acid; GCAO: 4-(N-(S-cysteinylglycylacetyl)amino)phenylarsonous acid; GSAO: 4-(N-(S-glutathionylacetyl)amino)phenylarsonous acid; PENAO: 4-(N-(S-penicillaminylacetyl)amino) phenylarsonous acid; VDAC: Voltage dependent anion channel; HKII: Hexokinase II.

## Competing interests

The authors declare that they have no competing interests.

## Authors' contributions

DP and JC performed the experiments and analysed the data. GGP assisted with the design of the yeast studies and performed some experiments. PJD and PJH conceived the study and wrote the manuscript. All authors approved the final manuscript.
